# Supplementary material for: Androgen-Independent Prostate Cancer Is Sensitive to CDC42-PAK7 Kinase Inhibition
Source: Biomedicines. 2022 Dec 30;11(1):101. doi: 10.3390/biomedicines11010101 (PMC9855385; doi:10.3390/biomedicines11010101)
Supplement: Supplementary file 1 [file biomedicines-11-00101-s001.zip › biomedicines-2067430-supplementary/biomedicines-2067430-supplementary-rev-HH.pdf]

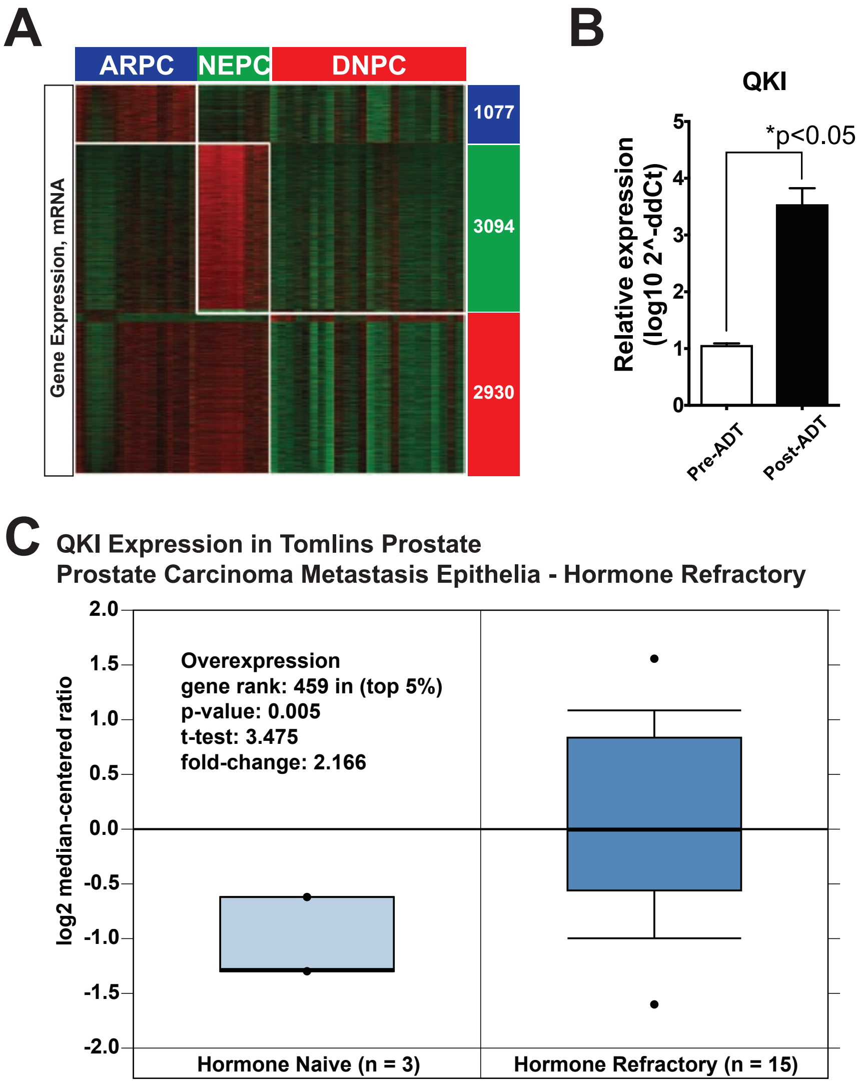

Figure S1. QKI mRNA levels in primary and metastatic prostate cancer tissues

A. Subtype-specific mRNA signatures for prostate cancer in WCM dataset. The figure shows the expression pattern of microRNA in relation to the molecular subtypes of prostate cancer (AR/NE/DN). Subtype-specific signatures were identified using multiple t-tests between the subtype of interest and the other subtypes, with a cut-off of $P<0.05$.
B. QKI mRNA qRT-PCR values in pre- and post-ADT primary prostate cancer tissues (n=3 per each). The figure shows the QKI mRNA levels measured using qRT-PCR in primary prostate cancer tissues before and after treatment with androgen deprivation therapy (ADT). The patients received 6-month bicalutamide 50mg monotherapy before undergoing radical prostatectomy.
C. QKI mRNA levels from DNA microarray results of metastatic prostate cancer tissues. The figure shows the QKI mRNA levels measured using DNA microarray analysis in metastatic prostate cancer tissues. The data was obtained from Oncomine.org and was originally published in Tomlins et al., Nat Genet. 2007

A Tumorsphere representative images

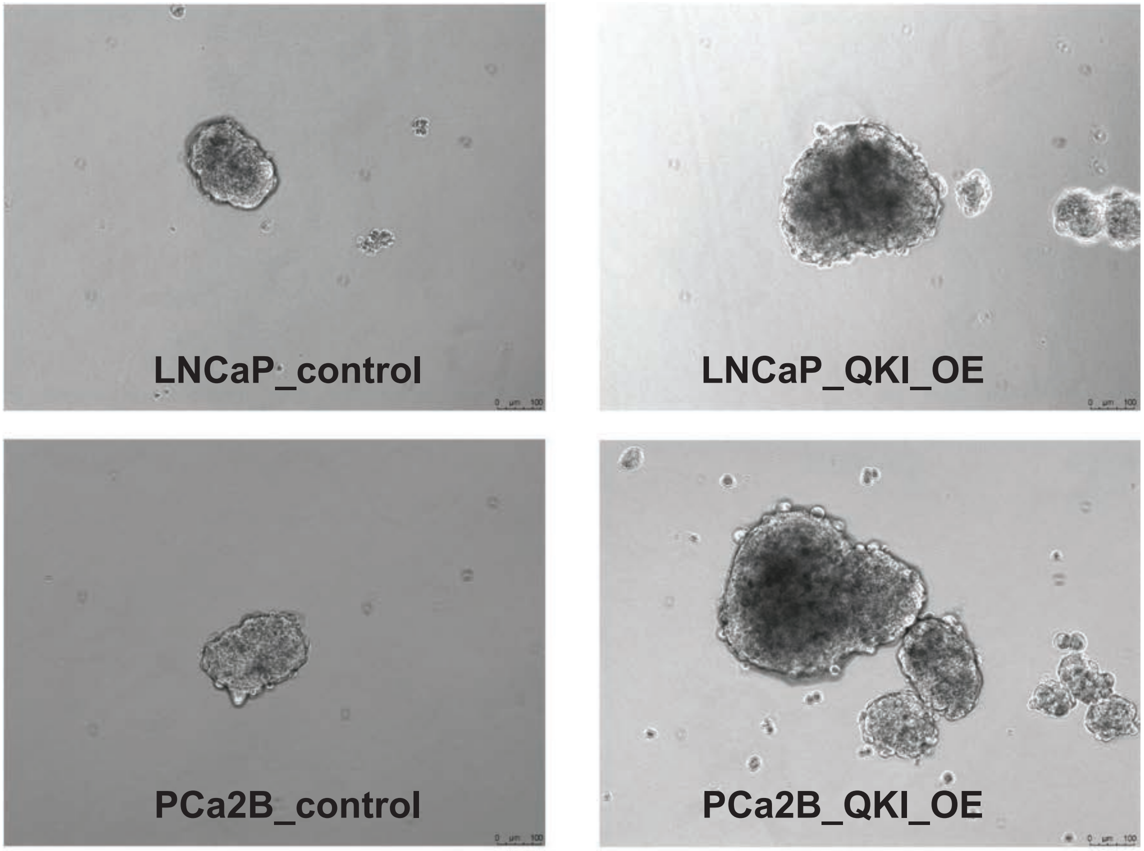

B 2D culture representative images

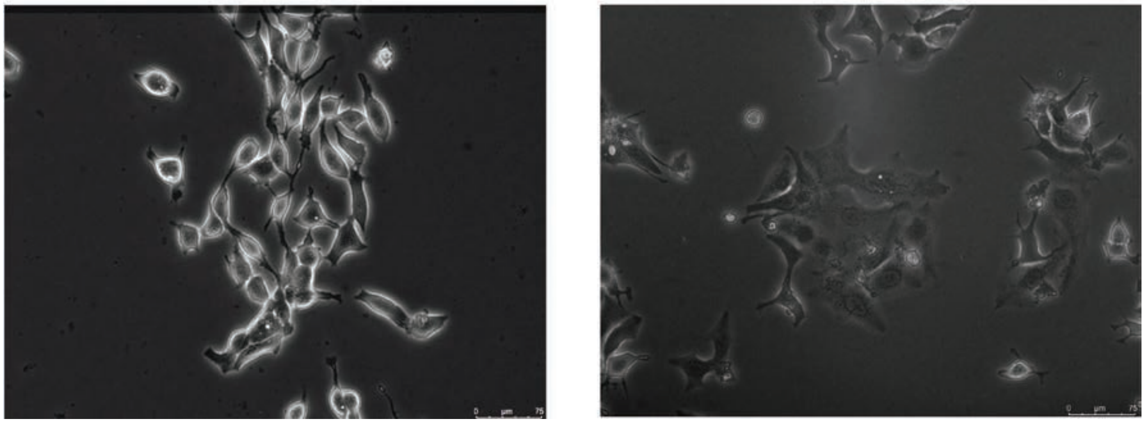

LNCaP_Control LNCaP_QKI_OE

Figure S2. CRISPR-mediated QKI overexpression and its effect on tumorsphere formation

A. Representative images of tumorsphere formation. LNCaP cells or PCa2B cells with control CRISPR and QKI_CE were cultured for 10 days in prostosphere cultivation media. QKI_OE refers to QKI overexpression achieved through CRISPR-knock-in-promoter activation.

B. Representative images of two-dimensional culture space.

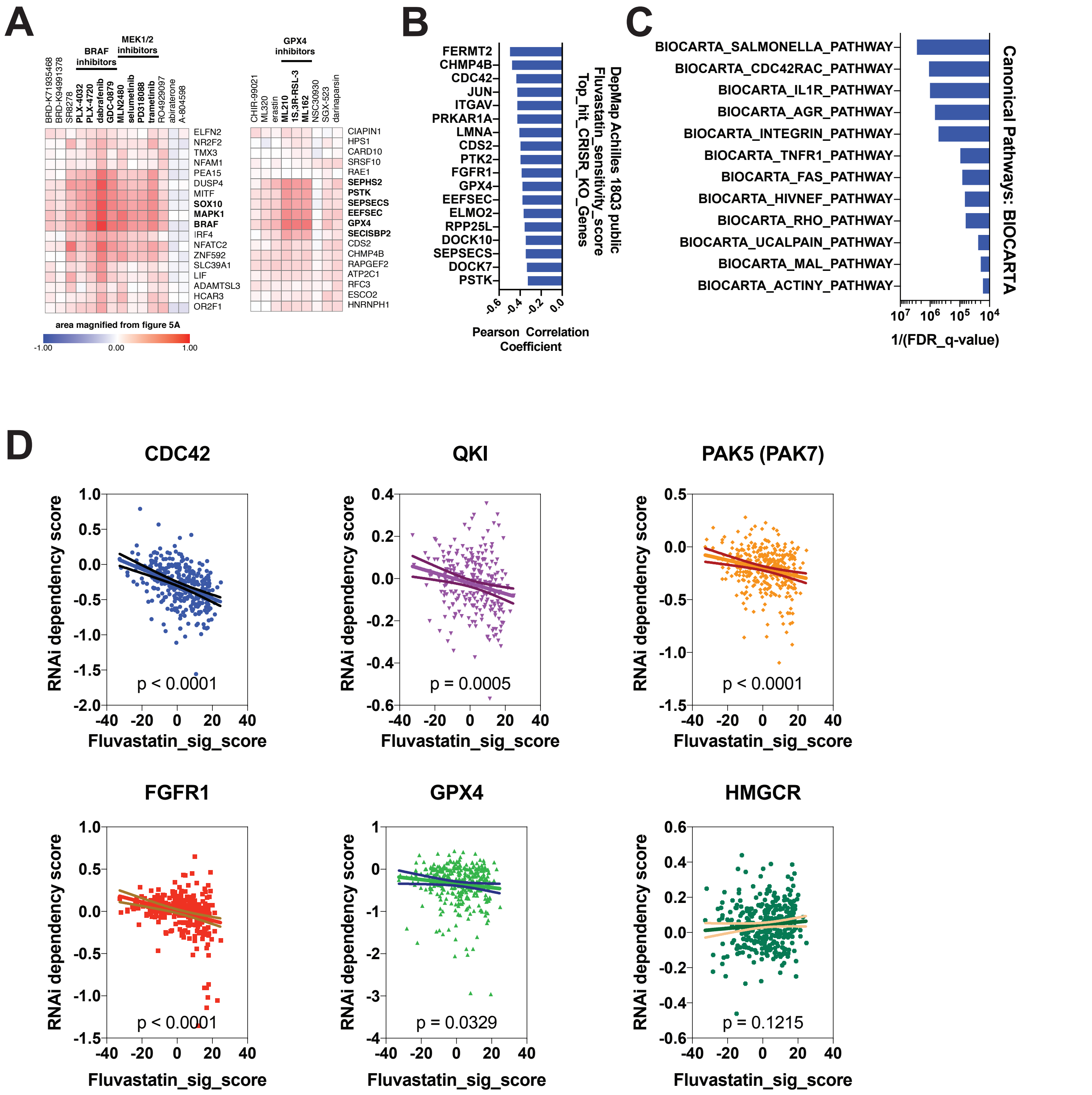

Figure S3. Fluvastatin sensitivity and its association with CRISPR knock-out sensitivity profiles

- **A.** Magnified view of the correlation matrix between drug sensitivity and genetic perturbation sensitivity (refer to Figure 5A for full matrix).
- **B.** Top 0.1% genes with CRISPR knock-out sensitivity profiles that positively associate with fluvastatin sensitivity gene scores. Gene expression and CRISPR knock-out library effect data were obtained from DepMap.org (DepMap Public 18Q3 release, CCLE). Genes significantly correlated with Fluvastatin sensitivity were used to generate scores based on the sum of their mRNA expression z-scores.
- **C.** Top canonical pathway genesets (from the BIOCART database) that are enriched in fluvastatin-sensitive cells compared to insensitive cells. The genesets were obtained from the Molecular Signatures Database.
- **D.** Correlation profile of CDC42, QKI, PAK5(PAK7), FGFR1, GPX4 and HMGCR RNAi sensitivity to fluvastatin sensitivity score. The plot shows the Pearson correlation coefficients for n=338 samples. The p-value is based on a two-tailed test. The slope indicates the linear regression best-fit values with 95% confidence intervals.

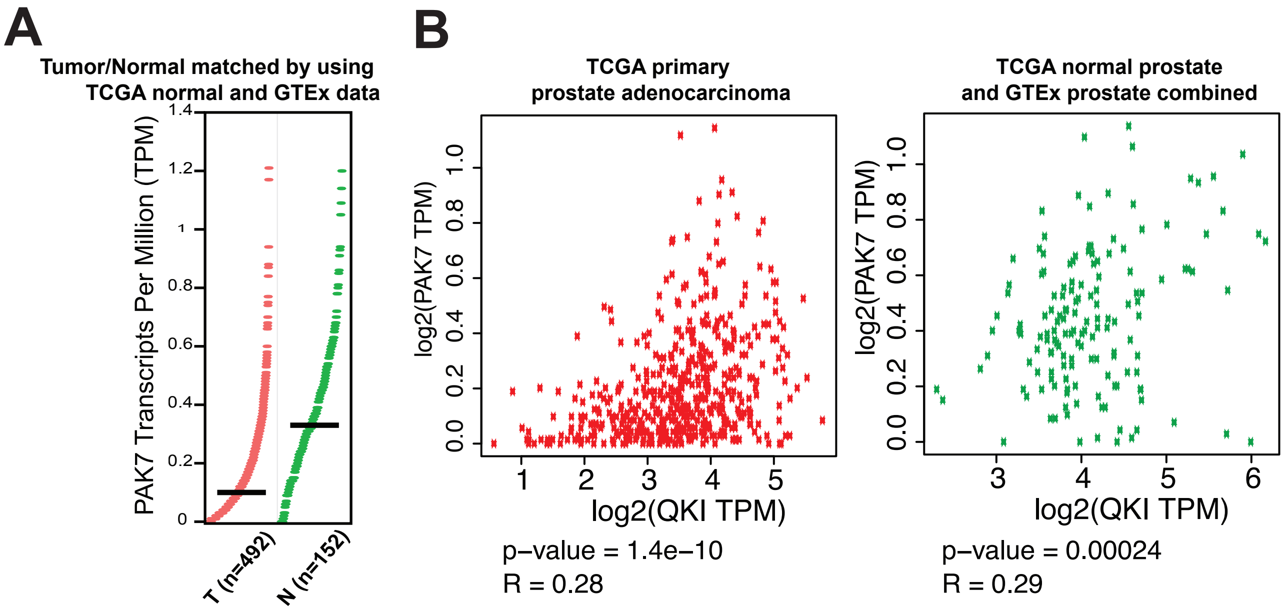

Figure S4. PAK7 mRNA expression and its correlation with QKI in prostate tissues

- **A.** Comparison of PAK7 mRNA expression between prostate tumors (red) and normal tissues (green). The bar represents the median expression. The data was assessed using the Gene Expression Profile Interaction Portal (http://gepia.cancer-pku.cn/). The original tumor/normal data are from TCGA and GTEx.
- **B.** Correlation of PAK7 mRNA expression with QKI in prostate tumors (left) and normal tissues (right).
